# Endoscopic ultrasound safety and efficacy in pediatric pancreatobiliary and gastrointestinal disorders: single-center retrospective trial

**DOI:** 10.3389/fped.2025.1684339

**Published:** 2025-09-23

**Authors:** Jiayu Zhang, Zhaohui Deng

**Affiliations:** Department of Pediatric Digestive, Shanghai Children’s Medical Center, Shanghai Jiao Tong University School of Medicine, Shanghai, China

**Keywords:** biliary tract diseases, endoscopic ultrasonography, gastrointestinal disease, pancreatic diseases, pediatrics

## Abstract

**Background:**

While endoscopic ultrasound (EUS) is well established as a standard diagnostic and therapeutic tool in adults, its use in children remains limited. This study assessed the indications, safety, and clinical impact of diagnostic and therapeutic EUS in children with pancreatobiliary and gastrointestinal disorders.

**Methods:**

We retrospectively analyzed pediatric patients with pancreatobiliary and gastrointestinal disorders who underwent EUS at our institution between January 2022 and June 2025.

**Results:**

Fifty-three EUS procedures were conducted in children with a median age of 9.6 ± 3.0 years. Indications for EUS included acute recurrent pancreatitis (*n* = 15), suspected chronic pancreatitis (*n* = 12), suspected choledocholithiasis (*n* = 7), obstructive jaundice (*n* = 5), pancreatic mass (*n* = 1), gastric mucosal lesions (*n* = 6), suspected esophageal lymphoma recurrence (*n* = 1), suspected autoimmune hepatitis (*n* = 1) and pancreatic pseudocyst (PPC, *n* = 5). ERCP was performed during the same anesthesia for all children requiring it post-EUS. Five patients underwent cystogastrostomy for symptomatic PPC with 100% technical and clinical success. The efficacy of EUS in guiding definitive therapeutic decisions and invasive interventions was 100%. Notably, no major complications occurred.

**Conclusion:**

EUS is safe and effective diagnostic and therapeutic modality in pediatric patients with pancreatobiliary and gastrointestinal disorders.

## Introduction

Endoscopic ultrasound (EUS) integrates an endoscope with an ultrasound transducer at its tip to enable high-resolution imaging of the gastrointestinal layers and adjacent organs. Since its introduction in the early 1980s, EUS has been widely adopted in the diagnosis and management of digestive diseases in adults, with well-established efficacy and safety (1–3). Although the first reported use of EUS in pediatric patients dates back to the 1990s (4), its adoption in pediatric patients remains limited; this is largely due to the low prevalence of pancreaticobiliary diseases and gastrointestinal tumors in children, device size constraints, and a shortage of skilled pediatric EUS practitioners (5). Despite these barriers, the European Society for Paediatric Gastroenterology Hepatology and Nutrition (ESPGHAN) recommends EUS for evaluating pancreaticobiliary disorders in children when conventional fails to provide a diagnosis. Indications include congenital esophageal stenosis, gastric varices, submucosal gastrointestinal lesions, and gastrointestinal duplication cysts (6). Nevertheless, current pediatric EUS studies are predominantly small-scale (5, 7–11) and primarily focus on diagnostic applications; as a result, data on therapeutic interventions remains limited. In this single-center, retrospective study, we analyzed the indications, clinical benefits, and risks of EUS in children, specifically evaluating the efficacy and safety of EUS-guided drainage of pseudocysts.

## Methods

### Patients

We collected the clinical data of children under 18 years old who underwent EUS at the Department of Pediatric Gastroenterology, Shanghai Children's Medical Center, Shanghai Jiao Tong University School of Medicine between January 2022 and June 2025. Data included demographic information, EUS indications, anesthesia methods, procedural details of EUS, outcomes, and complications.

### Diagnostic criteria

Pancreas divisum (PD) was diagnosed when EUS imaging along the long axis of the pancreas failed to reveal the “double-duct sign” and the pancreatic duct did not course toward the major papilla (12). Pancreaticobiliary maljunction (PBM) was diagnosed when the common channel length after the confluence of the pancreatic and bile ducts exceeded 5 mm (13). Pancreatic parenchymal and ductal changes were assessed using the Rosemont criteria (14). Ductal changes included ductal dilation, intraductal stones, hyperechoic ductal margins, and dilated branches. Parenchymal changes included calcification, hyperechoic foci with or without acoustic shadowing, honeycomb-like changes with or without lobular architecture, and cyst formation. These findings are categorized into four types of pancreatic abnormalities: “normal”, “indeterminate”, “suggestive”, and “consistent with” chronic pancreatitis. Gallstones are defined as hyperechoic foci with posterior acoustic shadowing. The clinical impact of EUS was scored according to the method described by Bjerring et al. (15): 0, no impact on diagnosis or management; 1, establishment of a definitive diagnosis or exclusion of suspected pathological conditions; 2, identification of novel clinically relevant findings that subsequently altered the patient management strategy; and 3, discovery of relevant findings that led to the adoption of an EUS-based therapeutic approach.

### EUS procedure

All EUS procedures were performed simultaneously by an EUS-trained pediatrician and an experienced interventional endoscopist specializing in adult gastroenterology. An adult-sized linear echoendoscope (EG-580UT; Fujifilm, Tokyo, Japan) with a frequency of 7.5 MHz was used to conduct a three-station examination of the stomach, duodenal bulb, and descending duodenum to visualize the biliopancreatic system and observe ductal and parenchymal changes.

The miniprobe (SP-900; Fujifilm, Tokyo, Japan) was utilized for assessing submucosal lesion under 2 cm. For EUS-guided fine-needle aspiration (FNA) biopsy, 19-G or 22-G needles (Boston Scientific, Marlborough, MA, USA) were employed. The puncture site was determined based on the EUS findings, with color Doppler used to avoid vascular structures. The FNA procedure was performed using a slow-pull technique. Three passes were performed for solid lesions, and the core specimens were assessed macroscopically for adequacy. Pancreatic pseudocyst (PPC) drainage was performed using a 19-G needle (Cook Medical, Indianapolis, IN, USA). The puncture path was optimized to achieve the shortest distance between the lesion and gastric wall; to further guide the procedure, color Doppler imaging was used once again to confirm the absence of intervening vessels. Following successful puncture, the cyst fluid was collected, followed by guidewire placement. The tract was subsequently established by a circular incision knife and/or balloon dilator. Stent placement was performed under endoscopic and fluoroscopic guidance. Subsequently, stent removal was performed after computerized tomography (CT) imaging confirmed cyst resolution.

### Following up

Pediatric patients undergoing EUS-guided drainage stent removal for pancreatic pseudocysts underwent telephone follow-ups every 3 months to evaluate cyst recurrence.

### Statistical analysis

All statistical analyses were performed using IBM SPSS Statistics for Windows, version 23.0 (IBM Corp., Armonk, NY, USA). Data are presented as mean (median as well as mean) ± standard deviation (SD), and as number (n) and percentage (%).

## Results

### Clinical characteristics

Of the 53 pediatric patients who underwent EUS between January 2022 and June 2025, 27 were males and 26 were females. The mean age was 9.6 ± 3.0 years (range 3–14 years), and the mean weight was 34.0 ± 18.0 kg (range 14–78 kg). The indications for EUS were as follows: acute recurrent pancreatitis (ARP) in fifteen cases, suspected chronic pancreatitis (CP) in twelve cases, suspected choledocholithiasis in seven cases, obstructive jaundice in five cases, pancreatic mass in one case, gastric submucosal lesion in six cases, suspected esophageal lymphoma recurrence in one case, suspected autoimmune hepatitis in one case and pancreatic pseudocyst in five cases. Diagnostic EUS was performed in 48 cases, whereas therapeutic EUS was performed in five cases. Complications were observed in two patients: one experienced bleeding at the puncture site during EUS-FNA, which was controlled with topical epinephrine application, and the other experienced perioperative infection following EUS-guided drainage, which resolved with antibiotic therapy. Among the 53 patients, 32 underwent procedures under general anesthesia (GA) and 21 under intravenous anesthesia. The details are summarized in Table 1.

**Table 1:** Study population and indications for endoscopic ultrasound.

Variable	EUS
No. of patients	53
Age at time of EUS, mean (SD), y	9.6 ± 3.0
Male, *n* (%)	27 (51.0%)
Weight, mean (SD), kg	34.0 ± 18.0
Indication for procedure, *n* (%)	
ARP	15 (28.3%)
Suspected CP	12 (22.6%)
Suspected choledocholithiasis	7 (13.2%)
Obstructive jaundice	5 (9.4%)
Pancreatic mass	1 (1.9%)
Gastric submucosal lesion	6 (11.3%)
Suspected esophageal lymphoma recurrence	1 (1.9%)
Suspected autoimmune hepatitis	1 (1.9%)
Pseudocyst drainage	5 (9.4%)
Diagnostic, *n* (%)	48 (90.6%)
Therapeutic, *n* (%)	5 (9.4%)
Complications, *n* (%)	2 (3.8%)
Infection	1 (1.9%)
Bleeding	1 (1.9%)
GA, *n* (%)	32 (60.4%)

EUS, endoscopic ultrasound; SD, standard deviation; ARP, acute recurrent pancreatitis; CP, chronic pancreatitis; GA, general anesthesia.

### Clinical impact of EUS

**Figure 1:**
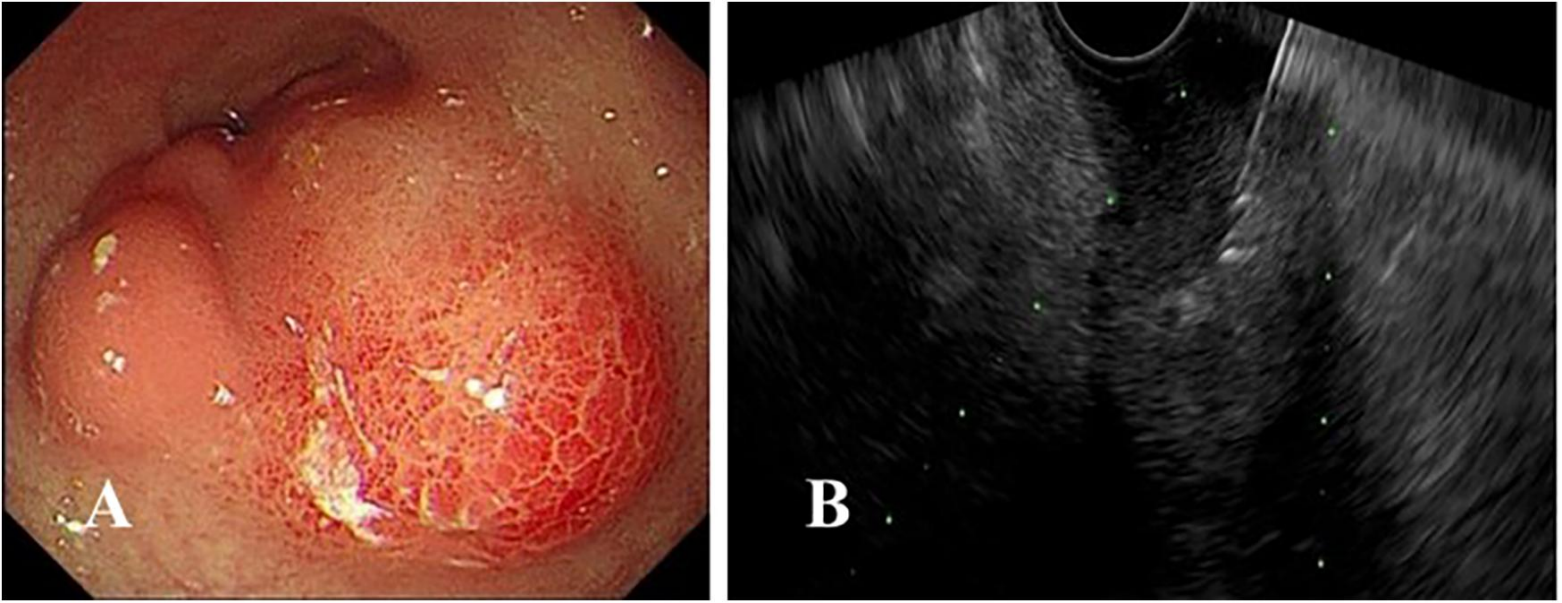
**(A)** Endoscopic findings revealing an irregular submucosal mass in the gastric antrum with overlying mucosa appearing congested and edematous; **(B)** endoscopic ultrasound-guided fine-needle aspiration was performed.

Among the 48 patients who underwent diagnostic EUS, fifteen presented with ARP. Of these, six were diagnosed with PD, five with CP, and four with idiopathic pancreatitis. Among the twelve with suspected CP, seven were confirmed to have CP, and five were diagnosed with PD. The five patients with obstructive jaundice were diagnosed with PBM. Among the seven cases with suspected choledocholithiasis, three were found to have normal findings, allowing for the avoidance of unnecessary endoscopic retrograde cholangiopancreatography (ERCP). In addition, ERCP was performed during the same anesthesia for all children requiring it post-EUS. EUS-FNA was performed in five patients, yielding definitive histopathological diagnoses in four cases, corresponding to diagnostic efficacy of 80%. One patient had inconclusive results owing to blood contamination in the sample. Final diagnoses included ectopic pancreas (Figure 1), lymphoma, inflammatory granulation tissue, autoimmune hepatitis and one undetermined case. In the latter, pre-EUS contrast-enhanced abdominal CT suggested a pancreatic mass; however, EUS confirmed its origin from the gastric muscularis propria, raising the suspicion of a stromal tumor. This finding played a critical role in surgical planning, and postoperative pathology confirmed the presence of a myofibroblastoma. The details are summarized in Table 2.

**Table 2:** Impact of endoscopic ultrasound.

Pre-EUS diagnosis	No.	Post-EUS diagnosis/No.	Clinical impact/No.	Treatment
ARP	15	PD/6	2/6	ERCP
		CP/5	2/5	ERCP
		Normal/4	1/4	Precluded need for ERCP
Suspected CP	12	CP/7	2/7	ERCP
		PD/5	2/5	ERCP
Suspected choledocholithiasis	7	Choledocholithiasis/3	2/3	ERCP
		Normal/3	1/3	Precluded need for ERCP
		PBM/1	2/1	ERCP
Obstructive jaundice	5	PBM/5	2/5	ERCP
Pancreatic mass	1	Lymphoma/1	1/1	Chemotherapy
Gastric submucosal lesion	6	Suspicious stromal tumor/1	1/1	Surgery
		Ectopic pancreas/3	1/4	Surgery for one case, follow-up for tree cases
		Gastric stromal tumor/2	1/2	follow-up
Suspected esophageal lymphoma recurrence	1	Inflammatory granulation tissue/1	1	Endoscopic esophageal dilation
Suspected autoimmune hepatitis	1	Autoimmune hepatitis/1	1	Prednisone and mercaptopurine combination therapy

EUS, endoscopic ultrasound; ARP, acute recurrent pancreatitis; PD, pancreas divisum; ERCP, endoscopic retrograde cholangiopancreatography; CP, chronic pancreatitis; PBM, pancreaticobiliary maljunction.

### Therapeutic EUS

**Figure 2:**
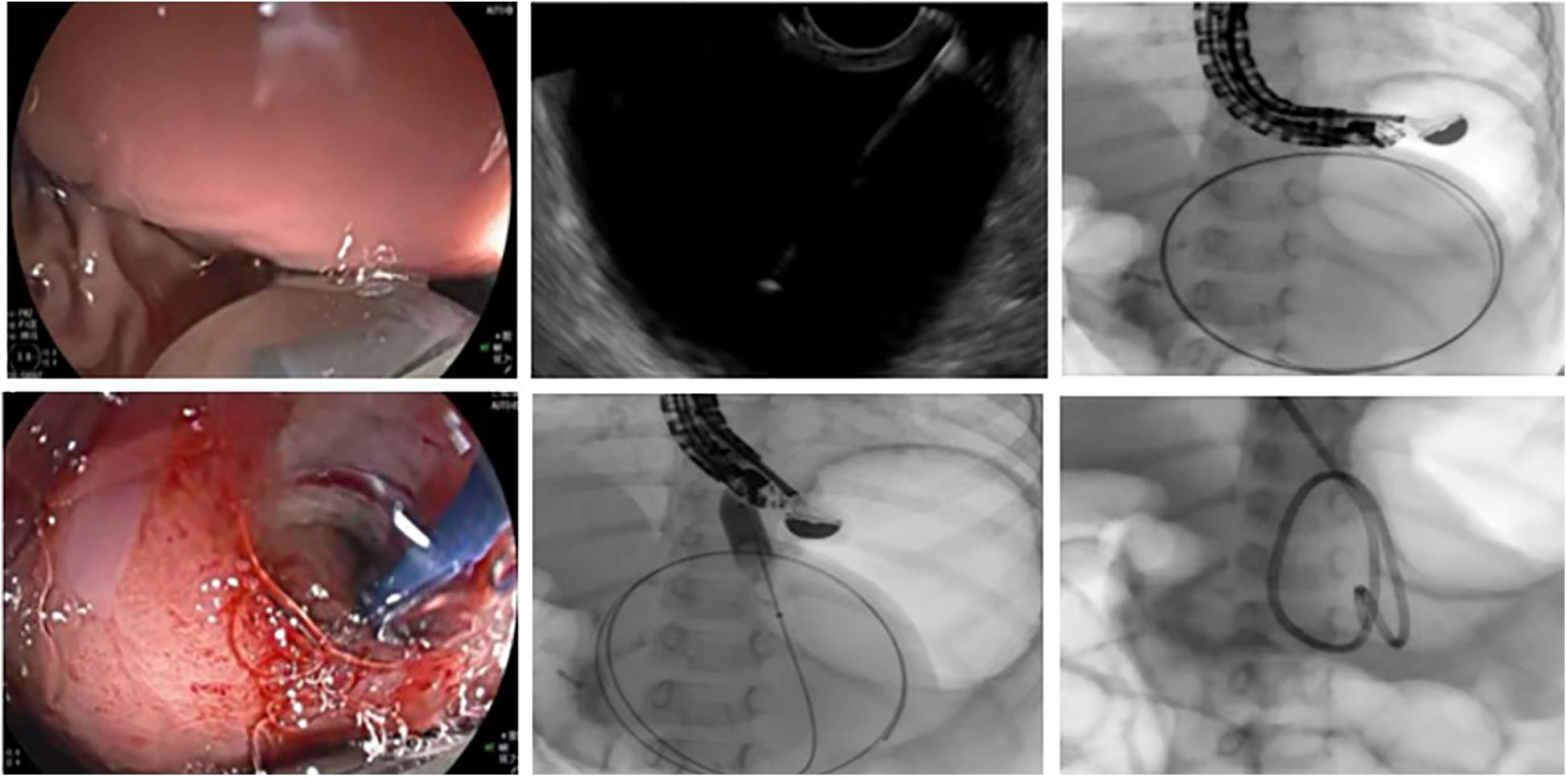
The procedure of endoscopic ultrasound-guided drainage for pancreatic pseudocyst.

A total of five therapeutic EUS procedures were performed on the PPC. The underlying etiologies included asparaginase-associated pancreatitis (AAP) in four cases following treatment for acute lymphoblastic leukemia and CP with PD in one case. The mean duration of pseudocyst persistence prior to intervention was 3.7 months (range: 1.5–6 months). Two patients underwent EUS-guided drainage following unsuccessful ERCP-based pseudocyst management. Double-pigtail plastic stents were successfully placed in four patients. In one case (Figure 2), stent placement failed due to the pseudocyst's proximity to the cardia, prompting the use of nasocystic drainage stent, which was removed after 12 days upon CT-confirmed cyst resolution. Among the four patients who received double-pigtail stent, three had their stents removed after 12 weeks following CT-confirmed complete resolution. In the remaining one case, the stent was removed at 4 weeks due to the small cyst size. During follow-up, one patient with AAP experienced asymptomatic 2 cm cyst recurrence 1 month post-stent removal, which resolved spontaneously within 3 months under conservative management. All procedures achieved technical and clinical success. Concurrent ERCP was performed in three patients during EUS-guided drainage: one with CP and pancreatic duct abnormalities and two with AAP (one with proximal pancreatic duct stricture and one with PD). The details are summarized in Table 3.

**Table 3:** Clinical characteristics of five pediatric patients with pancreatic pseudocyst.

Clinical data	Pt1	Pt2	Pt3	Pt4	Pt5
Age/sex	11/M	4/M	12/M	11/F	7/M
Etiology	CP with PD	AAP	AAP	AAP	AAP
Symptoms	Abdominal pain and vomiting	Abdominal pain	Abdominal pain	Abdominal pain and vomiting	Abdominal pain
Duration (months)	2	6	3	1.5	6
Size(cm*cm)	8.7*6.0	8.2*7.8	15*12	7.9*7.6	3.5*4.0
Location	Tail of the pancreas				
Needle size	19G FNA				
Single/double stents and size	Two, DPT stents 5Fr. ×5 cm	nasopancreatic drainage, 7Fr.	Two, DPT stents 7Fr. ×5 cm	Two, DPT stents 7Fr. ×5 cm	Two, DPT stents 7Fr. ×5 cm
Time of stent removal (weeks)	12	1	12	12	4
Complications	No	No	No	Infection	No
ERCP procedures	Pancreatic duct stone extraction; Dorsal pancreatic duct drainage			Sphincterotomy, pancreatic duct drainage	Sphincterotomy, Dorsal pancreatic duct drainage
Follow up after stent removal (months)	26	18	19	12	8
Resolution of pseudocyst in follow-up imaging	No recurrence				Recurrence after 1 month

CP, chronic pancreatitis; PD, pancreas divisum; AAP, asparaginase-associated pancreatitis; FNA, fine-needle aspiration; DPT, double pigtail.

## Discussion

The utilization of EUS in pediatric gastroenterology is gradually expanding, although experience in children remains limited compared to that in adults. In this study, we included 53 children aged 3–14 years, with biliary-pancreatic evaluation accounting for the majority of common indications (86.5%). While biliary microlithiasis accounts for 25%–75% of unexplained acute pancreatitis (AP) in adults (16, 17), the etiologies in pediatric cases differ significantly, often involving congenital pancreatobiliary malformations, genetic mutations, and medications (18). Among the fifteen cases of ARP in this cohort, EUS confirmed PD in six patients, CP in another five, and idiopathic pancreatitis in another four. Of the twelve suspected CP cases, seven were confirmed to have CP and five were diagnosed with as PD. Magnetic resonance cholangiopancreatography (MRCP) is a first-line non-invasive imaging modality for pancreatobiliary evaluation; however, its sensitivity is limited in children owing to its small duct caliber, respiratory motion artifacts, and intestinal fluid interference (19). Notably, none of the five PD cases in this study were definitively diagnosed using pre-EUS MRCP. Compared with MRCP, EUS provides superior spatial resolution, enabling detailed visualization of the pancreatic parenchyma and ductal structures. This capability is critical for detecting early-stage CP and clarifying idiopathic AP etiologies (20). Previous studies have reported CP in 11%–31% of pediatric ARP cases (21), with PD identified via EUS in approximately 14% of children with CP (22). In terms of biliary evaluation, EUS diagnosed PBM in five obstructive jaundice cases and excluded choledocholithiasis in three of seven suspected cases, avoiding unnecessary ERCP. EUS demonstrates 95%–100% accuracy for detecting microlithiasis (<3 mm) (11, 23), allowing children without EUS evidence of stones to avoid unnecessary cholecystectomy or ERCP. These findings underscore the pivotal role of EUS in the diagnostic and therapeutic management of pancreaticobiliary disorder in children.

EUS-FNA enables histopathological evaluation, particularly in cases where imaging alone yields inconclusive findings. Although gastrointestinal neoplasms are rare in the pediatric population, prior studies have identified pancreatic masses or autoimmune pancreatitis as the primary indications for EUS-FNA in children (4, 5, 24). In this study, EUS-FNA was performed in five patients with the following indications: pancreatic mass, gastric submucosal lesion, suspected recurrent esophageal lymphoma, and suspected autoimmune hepatitis; the procedure yielded a histological diagnostic rate of 80%, consistent with previously reported rates of 69%–88.1% (10, 25–27). One case complicated by post-procedural bleeding and blood-contaminated specimens, were subsequently diagnosed via surgical resection as an inflammatory myofibroblastic tumor, attributed to the lesion's intrinsic hypervascularity. Lesions exhibiting significant vascularity on Doppler imaging are generally considered unsuitable for EUS-FNA.

All five therapeutic EUS cases in our study involved symptomatic PPC with etiologies that differed from more commonly reported idiopathic and traumatic origins (28). In this cohort, 80% of cases were asparaginase-associated, while the remaining 20% were related to CP. When it came to treatment, two patients underwent EUS-guided drainage of the pseudocyst after ERCP with pancreatic proved unsuccessful. Three other patients required concurrent ERCP during EUS-guided drainage due to CP complicated by PD, proximal pancreatic duct stricture, and PD. Notably, none of the pseudocysts contained necrotic debris.

Regarding stent placement, double-pigtail stents were deployed in four cases, while a nasocystic stent was used in one patient due to the pseudocyst's proximity to the cardia. Notably, small-caliber plastic stents often fail in cases involving debris-containing PPC or walled-off necrosis (WON). In contrast, a recent retrospective study involving 32 pediatric patients with WON reported a 100% technical and clinical success rate using lumen-apposing metal stents (29), highlighting a promising alternative in complex cases. In our cohort, stents were removed between 1 and 3 months, with the longest follow-up extending to 26 months. Notably, no recurrence was observed, except for one asymptomatic 2 cm pseudocyst that resolved spontaneously within 3 months. Both technical and clinical success rates reached 100%, with only one perioperative infection, which was effectively managed with antibiotics. These findings are consistent with a systematic review (28), which confirmed the efficacy of EUS drainage in pediatric PPC, and reported 100% technical success and 10.9% complication rate, predominantly due to bleeding, stent migration, fever, and perforation. Collectively, these findings support the growing evidence that EUS drainage is a minimally invasive, highly effective, and low-risk technique for managing PPC in children.

Moreover, previous studies have demonstrated the safety and efficacy of EUS in pediatric populations (8, 9, 11, 30–32) with reported clinical impact rates of 35.5%–100% (5, 25, 33). In this study, EUS had a positive clinical impact in 100% of cases. Notably, patients diagnosed with conditions such as PD, CP, PBM, or choledocholithiasis using EUS underwent same-session ERCP under a single anesthesia session. Several studies have supported the benefits and safety of combined EUS-ERCP (8, 34), highlighting how pre-ERCP EUS facilitates rapid and precise pancreatobiliary evaluation, enhances diagnostic efficiency, and reduces the need for repeat sedation. Reported complication rates for pediatric EUS range from 1.96% to 3.8% (35), with most adverse events being mild and including pancreatitis, infection, bleeding, and perforation (36), consistent with adult risk profiles (37). In this study, we observed a slightly higher complication rate of 3.8%, consisting of bleeding and infection; this increase was likely due to the inclusion of higher-risk procedures such as EUS-FNA and EUS drainage, with all complications occurring during these procedures. Notably, diagnostic EUS alone exhibited risks comparable to those of standard endoscopy.

This study had certain limitations. First, this was a single-center retrospective study with a relatively small sample size. Second, the inherent selection bias of a tertiary care center may limit the generalizability of the study findings. Therefore, it remains necessary to conduct large-scale, multi-center prospective studies in the future for further validation. However, this study represents one of the more comprehensive evaluations of EUS in pediatric gastroenterology to date, encompassing EUS examinations, EUS-FNA, and therapeutic interventions.

In conclusion, EUS proved to be a safe and clinically impactful diagnostic and therapeutic tool for managing gastrointestinal disorders in children. Specifically, pre-ERCP EUS optimizes pancreatobiliary evaluation through rapid and precise assessment, while minimizing the need for redundant sedation. These findings suggest that EUS may be a valuable tool in pediatric gastroenterology; however, further multicenter studies are needed to validate and expand upon its role in clinical practice.

## Data Availability

The original contributions presented in the study are included in the article/Supplementary Material, further inquiries can be directed to the corresponding author.
